# Cooperative work and neuroeducation in mathematics education of future teachers: A good combination?

**DOI:** 10.3389/fpsyg.2022.1005609

**Published:** 2022-12-23

**Authors:** Marcos Procopio, Leandra Fernandes Procopio, Benito Yáñez-Araque, Raquel Fernández-Cézar

**Affiliations:** ^1^Mathematics Department, Faculty of Education of Toledo, University of Castilla-La Mancha, Toledo, Spain; ^2^Pedagogy Department, Faculty of Teacher Training and Education, Autonomous University of Madrid, Madrid, Spain; ^3^Department of Business Administration, Faculty of Law and Social Science, University of Castilla-La Mancha, Toledo, Spain

**Keywords:** cooperative learning, mathematics education, inclusive education, teaching, neuroeducation, future teachers, early childhood education

## Abstract

In order to respond to the new approaches in higher education, this paper presents the didactic experience of the implementation of a methodological approach based on cooperative learning and the literature review on neuroeducation in 53 students enrolled in the 3rd year of the degree in Early Childhood Education in the subject of Development of Logical-Mathematical Thinking and its Didactics II. The application of cooperative learning in combination with the literature review on neuroeducation aimed to teach future teachers how they should act with their future pupils (aged 3 to 6 years) with special educational support needs (SEN) at school. The design of the proposal to be used was also adjusted to the didactic resources available. The represented contents worked on were magnitudes, time, length, weight, and geometry. The data collected were the activities of the teacher training students (didactic proposals) adapted to the early childhood development stage. They were to be created, so that the main objective was that the undergraduate students would be able to solve the exercises as children would. The students were even challenged to make an exhibition in which they had to act as teachers and their classmates would be the children with SEN. The proposals were evaluated according to a rubric, with an emphasis on the students’ teachers’ awareness of students with SEN. To conclude, the degree of satisfaction of the student teachers with this methodology was high. This shows that learning neuroeducation and cooperative work makes future teachers know how to teach mathematics also to students with SEN.

## Introduction

The change that society has undergone throughout its history does not only involve the structures that represent the environment in which man has chosen to live. This change has been accompanied by a constant restructuring of the intellectual formation of its members in all areas, especially education or employment, by providing training programs to cultivate talent (Yáñez-Araque et al., 2021; Ruiz-Palomino et al., 2022). But for this change to continue to flow in an increasingly intense way, it is necessary to be concerned with the training of those who will educate future generations. Specifically, this work will deal with part of these people, the future teachers of early childhood education.

One of the concerns in the training of future teachers is precisely the difficulties they have with mathematics (Pañellas, 2016), given that some of them show these difficulties when they are students in their pre-university stages. Therefore, it is necessary to work with them to demystify the subject, alleviate their fears and give them didactic tools so that they can work with them, especially with children with special educational needs (SEN). In this sense, especially because teaching today involves challenges that go beyond how to educate. It is interesting to bring teachers closer to the findings of neuroeducation. These findings can help them to understand how mathematics learning in general develops in the brain, and to develop their proposals with an inclusive vision for all their students.

Teaching in accordance with the use of student-centered methodologies in their own training and skills that are known as innovative/active methodologies with the aim of making it possible to introduce logical-mathematical thinking to children with SEN requires at least an understanding of what neuroscience offers as an aid. Therefore, the low performance in Mathematics subjects (Dove and Dove, 2015) in Early Childhood Education teachers is not only justified by the lack of attraction they may feel toward mathematics, but in the lack of knowledge of how the cognitive process occurs and the limited experience with this subject provided by the didactic approach used. These are some of the justifications that may cause these teachers not to transmit accessible and sometimes understandable mathematics. Although some active methodologies such as flipped classroom (Dove and Dove, 2015) and cooperative learning (Swift, 2012) have been used with prospective early childhood teachers, there are no clear conclusions about the effect on their performance. Although in these previous works prospective teachers were taught with some active methodologies, they were not challenged to teach their peers as is they were children, playing the university students the teacher and pupils roles, which is the novelty of the present paper.

With all this in mind, the objective of this work is to develop teaching skills in mathematics education with a focus on cooperative work and neuroeducation literature research.

## Theoretical framework

Throughout the history of mankind, the teaching-learning process has undergone several significant changes that have evolved along with the needs of society. Thus, the teaching of mathematics has evolved in a way that is as intense as the transformation of everything that society has built. Both teaching and learning ceased to be accessible only to a select group, to become generalized learning and, moreover, essential for all components of society (Vegas and Ricardo, 2006).

With this evolution came the concern for certain members of society who were initially not considered in this teaching-learning process: people with SEN. Consequently, the training of future teachers has also had to evolve for them to be able to act in a scenario in which their students are diverse. Therefore, for mathematics teaching to be developed in an inclusive way, both for students with and without SEN, it is necessary for the training of future teachers to include new experiences involving appropriate methodologies for this, as well as knowledge of neuroscience in relation to the learning of mathematics.

To do so, it must be understood that the teaching of mathematics involves some problems, three of which can be highlighted: The first is the type of methodology used in the training of future teachers, which should enable them to familiarize themselves with the subject in an enjoyable way, so that they can pass it on to their pupils with an emotion of enjoyment. The second is whether they are taught how to teach it and make it accessible to an increasingly diverse student body. The third, but no less important, is that mathematical knowledge brings into play contents and procedures that involve: memory, attention, as well as very complex mental procedures such as organizing ideas, comparing, analyzing, reasoning, making decisions, following a structure, and complying with rules (Radford, 2006; Radford and André, 2009).

Regarding the first point, among the methodologies that can be used with the aim of making future teachers have a positive experience with mathematics, is cooperative learning (Placencia, 2015), which, due to its characteristics, seems to be a more conducive methodology to start with. As early as 1991, the National Council of Teachers of Mathematics (NCTM) noted experiences in which it was successfully developed in mathematics teaching and learning (Davidson and Kroll, 1991).

But before we start using cooperative learning, we should at least be aware of its characteristics, as it is often confused with group work (Domingo, 2008). However, it is important to clarify that they are two completely different ways of working with learners. Cooperative learning has several characteristics that differentiate it from group work (Johnson et al., 1999), for example, the communication between the components is based on trust, mutual support, and reciprocal support where it is verified that the members come to understand that all will succeed due to the synergistic nature of the task. Therefore, cooperative learning develops competences and skills such as communication, listening, decision-making and leadership. Group work does not involve the number of procedures of cooperative learning, moreover, cooperative learning is more than the development of working together with peers at university. According to Díaz-Aguado (2015), cooperative learning leads to the development of learning, not being a mere group collaboration.

As the name itself indicates, the first thing to consider is the collaboration that must exist between the components of the group. Since the success of the group depends directly on the contributions that each one can make and implies the social recognition of all the components (García et al., 2001, p. 38). It also depends on how one’s own work and that of others is organized. This leads to the development of new competences and skills such as the ability to relate to peers and to manage these relationships. Another relevant feature to bear in mind, is that cooperative learning, does not promote competition between the groups that are formed, or even between the components of the group. This builds positive interdependence (Herrada and Baños, 2018). The idea is that success does not belong only to one component, but that if one is successful, it means that all will be successful.

In contrast to competitive methodologies, through cooperative learning, students develop skills such as solidarity among group members. Each person becomes responsible for presenting new proposals to the group, which leads to another skill, which is that learning is obtained from living together with others (Pegalajar Palomino and Colmenero Ruiz, 2013).

On the other hand, working mathematics with students who have special needs requires that the training of future teachers goes to another level. It is necessary to know how they learn to be able to teach (Fernández Bravo, 2010; NAYEC and NCTM, 2013; Hernández-Perlines et al., 2016), as well as to know how the brain can retain knowledge. This implies that knowing certain bases of neuroscience, one of which is neuroeducation, is fundamental for future teachers to contemplate the teaching of logical-mathematical thinking in an inclusive way, making it suitable for their diverse students, including students with SEN.

Students with SEN do not only include children with learning difficulties, physical or intellectual disabilities, but also those with high abilities, whether academic, sporting, or artistic. With all of them it is necessary to use special methods and resources that contribute to their development (Fernandes Procopio et al., 2022), and it is necessary for future teachers to be aware of this, given that one of the problems pointed out in the lack of success in mathematics is inadequate teaching (Fernández Bravo, 2010). Considering the approach from the knowledge of the functioning of the learner’s brain, which is explained from neuroscience, would allow teaching to be adapted to all learners, and this includes learners with SEN.

Therefore, both the choice of methodologies to be used to teach future teachers and the knowledge of how learning is supported by neuroscience. This can give them the ability to understand that “special education pupils go through the same difficulties as children in mainstream education, but the difference lies in the fact that their cognitive processes of memory, attention and language are more marked, at least in terms of probabilistic thinking” (López-Mojica, 2013, p. 172).

As much as this work points to innovative methodologies, it is not a defense that learning can be measured by the novelty of the techniques and resources used, but neither can it deny the support that methodologies such as cooperative learning bring to the teaching-learning process of future teachers.

Neuroscience, coupled with new methods of brain research, can generate benefits for education (Ocampo, 2019). This research allows us to understand how the brain learns in a deeper way, which allows us to expand its potential (Roig, 2017). From the application of the neuroscience of learning to education arises neuroeducation which, unlike neuroscience, is concerned with understanding how the brain interacts in the acquisition of knowledge in the teaching-learning process. In other words, neuroeducation focuses on working memory, executive functions and the contribution of strategies that influence the teaching-learning process. For Días (2021) it is essential that teachers, in addition to knowing the neurobiological bases of learning such as memory, emotions and attention, understand how the brain works, as well as its correspondence with learning. In this way, the use of active methodologies that promote in students the development of autonomy, intelligence, and the use of technologies, contribute to improving learning conditions. These include cooperative/collaborative learning (Gonzáles and Abad, 2020).

Thanks to neuroeducation, it is possible to understand the interaction that takes place in the brain now of learning. This makes it possible, for the future teacher, to know what types of methodologies he/she could use to make the learning of his/her students more effective. In this way, neuroeducation and cooperative learning can be used as a tool to support mathematics education.

## Methodology

The participants were 53 students, 51 of whom attended the classes regularly, all of them female, students of the 3rd year of the degree in Early Childhood Education and enrolled in the subject Development of Logical-Mathematical Thinking and its Didactics II, in a Spanish university.

The students were chosen for two reasons: first, because they are enrolled in a subject that represents the key point for them, where they always encounter the difficulties of how to teach mathematics and how to know if their students are learning. And second, to help them decide which methodological path they should follow as future teachers in mathematics classes.

As a procedure, it was decided to work with the 10 existing base groups so that they could satisfactorily and adequately develop the contents and objectives of the subject. At the end of each theoretical block, they were asked to develop a teaching plan related to a content, and to implement it with the didactic resources available in the Didactics laboratory. These resources were Cuisenaire rods, hydrostatic balance, among others. Each of the blocks were named in a way that represented all the content worked on during the 4-month period, as shown in Table 1.

**Table 1:** Distribution of the blocks in the subject Development of Logical-Mathematical Thinking and its Didactics II.

Blocks
**The construction of magnitude in children:**
1. Measurement
2. Length
3. Mass
4. Time
5. Extensive additive
6. Intensive or non-additive
**Geometry:**
7. Similar spaces
8. The construction and structuring of space
9. Spatial and plane shapes
10. Van Hiele adaptation to early childhood education

The blocks were structured, in such a way that one or more of the contents, presented in the teaching guide could be worked on in each, i.e., in block 1 all the contents related to measurements were worked on. To carry out the work, a structure was proposed that was analyzed and modified by the participants until the one shown in Table 2 was reached. The aim was for the work guide to be a living document that could be adapted to the needs of each practice and students’ group.

**Table 2:** Didactic guide used for the development of the internships.

**Title**
It should be creative and motivate students to want to take part in projects
**Introduction**
Justification of the proposal
**Theoretical framework**
The theoretical framework may contain both old and mainly recent authors
**Objectives**
Programming objectives
**Contents**
Contents of the program
**Recipient**
It will indicate the learners targeted and describe the difficulties that may be encountered in classes for them.
**Didactic proposed**
This should contain all the activities you intend to develop in class in an explained way and how you will work with the students.
**Curricular adaptations for SEN**
**Teaching strategies**
Here you should explain how you are going to develop what you set out to do in your class and list how you are going to do it.
**Bibliography**

As a third prompt, they were to investigate what neuroeducation has to say about how pupils with special needs learn mathematics in their early childhood class.

Ten practices were proposed, one for each group, in which the future teachers had to prepare activities adapted to the ages of the chosen pupils (between 3 and 6 years old), as well as propose curricular adaptations for pupils with SEN. The contents were: magnitudes, time, length, weight, and geometry. All the practices were followed by group tutorials with the aim of checking the degree of scope and adaptation of the approaches to the ages of the infant education pupils. This ensured the degree of theoretical learning of mathematics by the future teachers.

**Figure 1:**
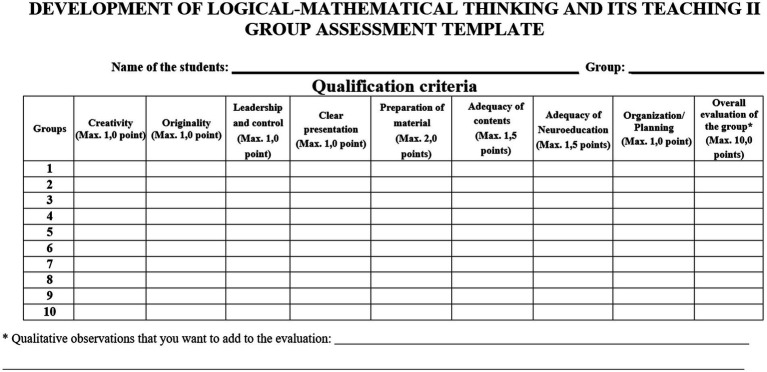
Group evaluation rubric.

The evaluation of the didactic proposals was carried out according to the criteria presented in Figure 1.

## Results

Table 3 shows the practices carried out during the course in the subject Development of Logical-Mathematical Thinking and its Didactics II, aimed at students with special educational needs.

**Table 3:** Lessons developed for children with special educational needs.

Practice	SEN	Topic	Score
Learning with Pinochio	Intellectual disability	Measurements	6,4
Hansel and Gretel: lighter or heavier	Autism spectrum disorder (ASD)	Mass	6,5
Nemo and the geometry of the ocean	Attention deficit hyperactivity disorder (ADHD)	Geometry	7,0
Dora the explorer and the geometric jungle	Attention deficit disorder	Geometry	6,8
Alice in the land of clocks	Dyscalculia	Time	7,2
Turbo and the race of the champion	High abilities	Length	7,1
Tadeo Jones and the geometric treasure	Intellectual disability	Spatial and plane figures	6,7
Matheolympics	ASD: Autistic spectrum disorder	Magnitude length	9,5
Dancing with numbers and geometric figures	ADHD: Attention deficit and hyperactivity disorder	Geometric figures	9,1
The flea market	High abilities	Numbers	9,3

Of the 10 groups that carried out the research, three stood out for the way they implemented neuroeducation knowledge in their classes. Table 3 presents the 10 practices, from which the three highest scoring practices were selected. These three practices correspond to the three groups that were most successful in developing and presenting their lessons using neuroeducation, as proposed in the written document prepared for each practice. These three groups made use of neuroeducation as an attempt to find new directions for the development of students with SEN.

In the first practice, Mathematics, the prospective teachers developed a lesson in which they had to point out the fundamentals of neuropedagogy and the methodology they were going to use to teach the magnitude of length. The lessons were conducted in an inclusive way, although with the great challenge of integrating students with SEN into the group. For Sales et al. (2015) it is worth mentioning that pupils with SEN are different from those with learning difficulties, given that the origin of the difficulties for the former is neurological. In terms of treatment, learning difficulties can be treated more easily, while students diagnosed with SEN require specific methodologies, depending on the degree of difficulty diagnosed, for the results of their treatment to be satisfactory. Autism spectrum disorder is a group of neurodevelopmental disorders that have in common both clinical expressions and the following characteristics: difficulties in reciprocal social interaction, difficulties in comprehension, expression and verbal communication and repetitive, restricted, and stereotyped behaviors (Artigás Pallarés and Narbona, 2011; Salvadó et al., 2012; Lozano et al., 2013).

To bring the activity closer to these students, one of the activities proposed was an assembly, in which both moments in which the student with special educational needs could remain in the group and moments in which they could not, according to their will, were proposed, offering them the materials they were working with so that they could familiarize themselves with the group or individually, respectively. Inclusive skills approaches and activities that aim to teach, using appropriate methodologies and understanding children’s brain development, promote learning. Studies such as that of Garnica et al. (2013) indicate that these pupils can develop an understanding of notions of length.

The next group worked with numbers and geometric figures. It could be observed that they not only sought to work on mathematical concepts, but also to apply them in different situations and everyday activities, as well as to develop intuition and social values through their experiences. They used these strategies to attract the attention of students with ADHD in a different way. According to Miranda et al. (2012) the difficulties presented by students with ADHD are related to impulsivity, restlessness, or inattention with respect to mathematical ability.

On the other hand, the future teachers also chose geometry because of its proximity to reality from an early age, thus keeping the attention of pupils with ADHD, as this part of mathematics is related to space and can be found in most of the materials around them, allowing them to work with exploration and observation of the environment, and facilitating the development of logical-mathematical thinking, in this case spatial.

However, the future teachers, expressed in their presentation and demonstrated with the activities that they considered of vital importance for working on mathematics with children with ADHD (although it is fundamental for all types of pupils), that mathematics will allow them to obtain an integral development, because in addition to working on mathematical concepts, other knowledge belonging to other contexts can be acquired. For example, the activities designed by this group in this study were aimed at working on mathematics, but also on other aspects, such as attention, socialization, respect, emotions, etc. This is in line with various authors (Yoo et al., 2014; Molina and Martínez-González, 2015; Johnstone et al., 2017) who work with neuroscience and report that improvements are found in the cognitive and emotional processes of students with ADHD.

In the last practice presented in this text, the future teachers chose to work with numbers, titling their exhibition The flea market. They sought to develop the concepts of number and simple addition and subtraction operations with students with High Abilities. They opted for classes with diversity, considering that children with high abilities get along better if they work in groups with other pupils and in a playful way. In this type of activity, the proposal is that the other children develop empathy as well as mathematics, and those with special educational needs reinforce their companionship by helping their peers, which would be a kind of motivation for the highly able pupils.

For Olszewski-Kubilius and Carenbach (2012), when highly able students understand that their success depends on hard work in school, they will do it. This implies that if teachers provide challenging lessons to these students, this will motivate them to continue. Motivation is an aspect of learning that has been explained from neuroscience, as well as its applications in education (Herce-Palomares and Abellán-Civera, 2018). Furthermore, these authors recognize that high abilities do not guarantee academic success or success in adulthood (Herce-Palomares and Abellán-Civera, 2018).

## Discussion

The teacher’s task always follows the same direction: to reflect on the objectives, contents, competencies, skills, and the material to be used in the classroom. However, the future teacher needs to go beyond what is known as the teaching task, he/she needs to value the interaction that the brain develops now of learning. This is what some authors defend, such as Poma and Castillo (2022), who go so far as to affirm that neuroeducational principles and factors are indispensable in the teaching-learning process of mathematics. If we add that the group of students present SEN, the knowledge of these neuroeducational principles becomes even more necessary.

The results obtained indicate that in practice with students with Autistic Spectrum Disorder (ASD), they can benefit from learning based on observation and methodologies for inclusion to occur (Torres, 2016). Students with ASD are also able to learn from their already lived experiences, applying neuroeducation and active methodologies. With the support of neuroeducation it is possible to adapt the organization of spaces that enable the development of skills and abilities to learn with the use of games, flavors, sounds, colors, and shapes, i.e., from sensory perception.

Regarding students with attention deficit hyperactivity disorder (ADHD), considered the most common neurobiological disorder (Rodillo, 2015), the future teachers developed a practice with their own didactic material that enables the teaching-learning process in these students. Factors such as involvement and goals achieved can generate well-being and attitudes that lead to the development of a positive psychology in people with ADHD (Newark et al., 2012). Among the elements for developing well-being are cooperative work, task involvement, meaning and goals (Seligman, 2019). In line with these elements, the future teachers chose geometry because of its proximity to objects close to the everyday life of students with ADHD and thus to work mathematics on aspects of attention, socialization, respect, emotions, and interaction.

Finally, the range of students with SEN also includes those with high abilities. These are characterized by intelligence, creativity, and motivation with the task, in addition to the emotion that is present in high learning abilities. From the perspective of neuroeducation, students in general may present learning problems that may be generated by a lack of motivation and interest (Días, 2021). Therefore, it is necessary for future teachers to know that students with high abilities develop a different cognitive processing, which involves acting and intervening on their attention and motivation, associating them with emotional principles that must be considered in the school context (Luque et al., 2016). In practice, future teachers used elements of neuroeducation to work with students with high abilities. Activities were proposed to reinforce motivation, interest, and their abilities such as creativity and intelligence, through cooperative work. Through the development of group, individual or paired projects, these students addressed the needs of their highly able pupils. In addition, the future teachers considered the emotional processes of students with high abilities, such as the small changes that can make personal adjustments that influence the improvement of the academic performance of these students (Rivera et al., 2014).

## Conclusion

The main contributions of this exploratory educational experience, carried out with future female early childhood education teachers, is the evidence that cooperative learning, in combination with neuroeducation research, makes these students prepare their own teaching materials with SEN awareness. By using cooperative learning, we have been able to provide the future teachers with a practical contact with mathematics, as well as the awareness that the students they will encounter in their classrooms will be diverse and their success as teachers involves learning from all of them. Therefore, their own research will be necessary to promote the learning of mathematics in all of them. In addition, this approach has allowed us to provide them with knowledge of neuroscience and they have been able to apply all of this to their teaching proposals for dealing with pupils with special educational needs.

The much talked about quality education that we all desire must be addressed from the initial training of teachers and must be approached from an inclusive perspective. This is one of the greatest challenges we have in teacher training, to train teachers capable of changing and modifying content, approaches, structures, and strategies, with a common approach that includes all children of the corresponding age and with the conviction that it is the responsibility of the general system to educate all children (Right to Education Initiative, 2019).

In mathematics, we have seen a significant qualitative leap in the motivation, interest, creativity and learning of future teachers. In addition to understanding the development of children’s logical-mathematical thinking, they have been able to understand that all children have the right to learn and can do so if, as teachers, we provide them with an experience appropriate to their level and educational needs.

Finally, it is important to implement methods in Mathematics Education that focus on cooperation, manipulation and adaptation of teaching materials and resources, making them inclusive and accessible to all students. There are no limits to learning, what there are barriers, and this is what future teachers must consider when teaching mathematics. From this perspective, their teaching role should be oriented toward reducing barriers to learning, offering inclusive educational proposals around mathematics and adequate support for their needs. This is what we want to call here the teacher’s awareness in welcoming diversity.

## Data availability statement

The raw data supporting the conclusions of this article will be made available by the authors, without undue reservation.

## Ethics statement

This research project has been granted full approval by the Social Research Ethics Committee of the University of Castilla-La Mancha under reference CEIS--634122-B5K2. The patients/participants provided their written informed consent to participate in this study.

## Author contributions

MP, LFP, BY-A, and RF-C: conceptualization, validation, formal analysis, investigation, and visualization. MP and LFP: methodology and writing—original draft preparation. MP: resources. LFP: data curation. BY-A and RF-C: writing—review and editing, supervision, and project administration. All authors contributed to the article and approved the submitted version.

## Funding

This project has been carried out under financial support of the Mathematics Department of Castilla La Mancha University.

## Conflict of interest

The authors declare that the research was conducted in the absence of any commercial or financial relationships that could be construed as a potential conflict of interest.

## Publisher’s note

All claims expressed in this article are solely those of the authors and do not necessarily represent those of their affiliated organizations, or those of the publisher, the editors and the reviewers. Any product that may be evaluated in this article, or claim that may be made by its manufacturer, is not guaranteed or endorsed by the publisher.
